# Novel endoscopic approach for safe and effective resection of duodenal neuroendocrine tumor

**DOI:** 10.1055/a-2440-6362

**Published:** 2024-11-08

**Authors:** Shinpei Minami, Shuhei Fukunaga, Michita Mukasa, Daiki Ohzono, Hiroshi Tanaka, Tomoyuki Nakane, Hidetoshi Takedatsu

**Affiliations:** 1 26333 Division of Gastroenterology, Department of Medicine, Kurume University School of Medicine, Kurume, Japan

A 76-year-old woman presented with a 10-mm duodenal neuroendocrine tumor (d-NET) located in the duodenal bulb and confined to the submucosa ( Fig. 1 **a** ). Conventional endoscopic mucosal resection (EMR) or endoscopic submucosal dissection (ESD) carries high risks of perforation and of obscuring the vertical margin. We have devised a method ( Video 1 ) to provide an equivalent treatment option in facilities where the full-thickness resection and closure device is unavailable.

**Fig. 1:**
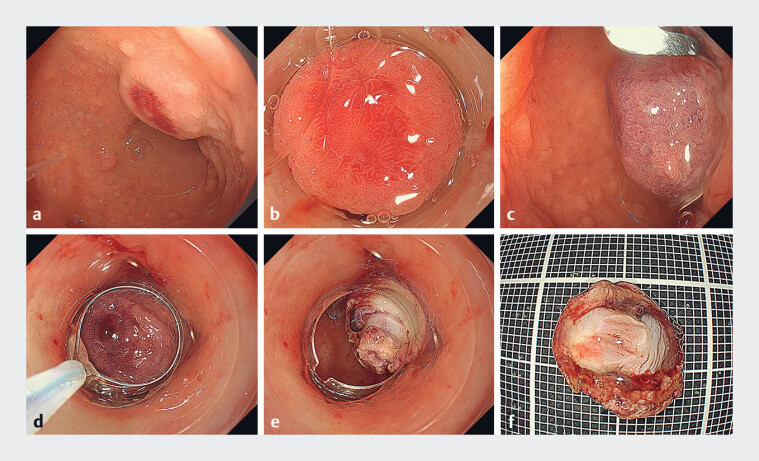
Cap-assisted endoscopic mucosal resection of a duodenal neuroendocrine tumor (d-NET) with an over-the-scope (OTS) clip. **a** The 10-mm d-NET. **b** Confirmation that the lesion could be suctioned into the distal cap attachment. **c** An OTS clip is deployed directly beneath the lesion, resulting in a pseudopolypoid elevation. **d, e** The lesion is resected by cap-assisted endoscopic mucosal resection with the OTS clip effectively preventing perforation. **f** Endoscopic full-thickness resection is achieved.

Novel cap-assisted endoscopic mucosal resection of a duodenal neuroendocrine tumor (d-NET) with an over-the-scope (OTS) clip.Video 1

Initially, a GIF-XZ1200 endoscope (Olympus, Tokyo, Japan) equipped with a distal cap attachment was employed to confirm that the lesion could be suctioned into such an attachment ( Fig. 1 **b** ). The endoscope was then switched to a GIF-2TQ260M (Olympus), fitted with an over-the-scope (OTS) clip system (10 mm; Ovesco Endoscopy); the clip was deployed directly beneath the lesion, resulting in a pseudopolypoid elevation of the lesion ( Fig. 1 **c** ). The endoscope was switched back to the GIF-XZ1200 with a distal attachment (MAJ-290, Olympus) and a snare (SD-221-L25) was positioned ( Fig. 1 **d** ). The elevated lesion was resected under full suction into the attachment. Endoscopic full-thickness resection was achieved, with the OTS clip effectively preventing perforation ( Fig. 1 **e, f** ). Histopathological examination confirmed an 8-mm NET (grade 1) limited to the submucosal layer, with negative margins, and without lymphovascular invasion ( Fig. 2 **a, b** ).

**Fig. 2:**
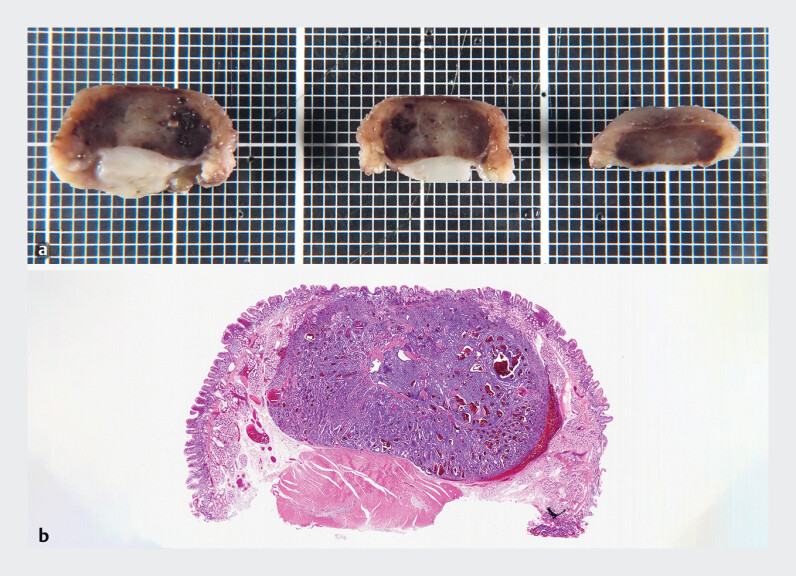
**a** Resected specimen. **b** Hematoxylin–eosin staining (low magnification).

In Japan, where the full-thickness resection device is not available, the EMR with OTS clip technique, termed EMRO, offers a promising method for treating d-NETs 1 . However, certain cases may pose challenges, particularly in those of snare resection. In the present case, using full suction with a transparent cap fitted over the OTS clip facilitated successful snaring, demonstrating the simplicity and reliability of this technique. This EMROC method is less costly than using the full-thickness resection device for lesions less than 10 mm, while effectively providing full-thickness resection for d-NETs.

Endoscopy_UCTN_Code_TTT_1AO_2AG_3AF

## Notes

Endoscopy E-Videos https://eref.thieme.de/e-videos
*E-Videos* is an open access online section of the journal *Endoscopy* , reporting on interesting cases and new techniques in gastroenterological endoscopy. All papers include a high-quality video and are published with a Creative Commons CC-BY license. Endoscopy E-Videos qualify for HINARI discounts and waivers and eligibility is automatically checked during the submission process. We grant 100% waivers to articles whose corresponding authors are based in Group A countries and 50% waivers to those who are based in Group B countries as classified by Research4Life (see: https://www.research4life.org/access/eligibility/ ). This section has its own submission website at https://mc.manuscriptcentral.com/e-videos .
